# The Immune Activity of PT-Peptide Derived from Anti-Lipopolysaccharide Factor of the Swimming Crab *Portunus trituberculatus* Is Enhanced when Encapsulated in Milk-Derived Extracellular Vesicles

**DOI:** 10.3390/md17050248

**Published:** 2019-04-26

**Authors:** Bao-Hong Lee, Bo-Rui Chen, Cheng-Ting Huang, Cheng-Hui Lin

**Affiliations:** 1 Division of Hematology and Oncology, Department of Internal Medicine, Taipei Medical University Hospital, Taipei 11031, Taiwan; f96b47117@ntu.edu.tw; 2 Department of Traditional Chinese Medicine, Department of Internal Medicine, Taipei Medical University Hospital, Taipei 11031, Taiwan; 3 Doctoral Degree Program in Marine Biotechnology, National Taiwan Ocean University, Keelung 20224, Taiwan; porrychen0601@gmail.com; 4 Doctoral Degree Program in Marine Biotechnology, Academia Sinica, Taipei 11529, Taiwan; 5 Department of Aquaculture, National Taiwan Ocean University, Keelung 20224, Taiwan; cthuang@mail.ntou.edu.tw

**Keywords:** antimicrobial peptide, *Portunus trituberculatus*, Anti-lipopolysaccharide factor, extracellular vesicles, THP-1 cells, immune stimulator

## Abstract

PT-peptide is derived from the anti-lipopolysaccharide factor of the swimming crab *Portunus trituberculatus*. The peptide, consisting of 34 amino acids, contains a lipopolysaccharide binding domain. In this study, we investigated the effect of PT-peptide encapsulated in raw milk-derived extracellular vesicles (EVs), designated as EVs-PT peptide, on immune regulation. The results showed that raw milk-derived EVs efficaciously delivered the PT-peptide into monocytes and elevated immune activity, including reactive oxygen species level, superoxide anion production, and phagocytosis. PT-peptide and EVs-PT peptide also elevated the secretion of cytokines, such as interferon-γ, interleukin-6, and tumor necrosis factor-α in human monocytic THP-1 cells. These results suggest that the PT-peptide could be developed as an immune stimulator.

## 1. Introduction

Antimicrobial peptides (AMPs) are an important component of the natural defenses of most living organisms against invading microorganisms and are relatively small (<100 amino acids), cationic, amphipathic peptides [1]. These peptides exhibit broad-spectrum activity against a wide range of microorganisms, including Gram-positive and Gram-negative bacteria, protozoa, yeast, fungi, and viruses. A few peptides have also been found to be cytotoxic to tumor cells [1,2,3,4]. Anti-lipopolysaccharide factor (ALF) is one type of AMP and plays an important role in the defense system of crustaceans [5,6,7,8,9,10]. ALFs typically share a conserved three-dimensional structure, consisting of three α-helices packed against a four-stranded β-sheet, and contain a putative lipopolysaccharide binding domain [5]. ALFs not only have broad-spectrum antimicrobial activity against gram-positive and negative bacteria [6,7,8,9] but also can bind and neutralize lipopolysaccharide [10]. Many ALFs have been found and characterized in crustaceans [6,7,8,9,11] and a variety of ALF isoforms have been reported to coexist in one organism. For example, at least eight different isoforms of ALFs have been identified in swimming crab, *Portunus trituberculatus* [9]. Most previous studies about the role of ALFs in crustaceans have investigated in vitro antimicrobial activity and characterization of ALF gene expression following microbial challenge or infection [6,7,8,9]. Recently, it has been reported that ALF can reduce white spot virus propagation [12,13]. Therefore, ALFs not only have in vitro antimicrobial activity and directly kill pathogens, but also appear to function in modulating immunity and protecting the host from infection.

Gastric acid, enzymes, and immune cells destroy or eliminate many active protein drugs. In order to maintain and enhance the activity of these protein drugs, the development of coating technology has become an important issue at present. Currently, the most preferred drug delivery systems are liposomes and polymeric nanoparticles. Liposomes are synthetic vesicles with a phospholipid membrane that self-assemble into various sizes and shapes in an aqueous environment [14]. Polymeric nanoparticles are used in drug delivery systems to entrap, encapsulate, or attach drug molecules [15]. Both delivery systems have been used to deliver different types of drug molecules, including anti-cancer drugs, anti-fungal drugs, and analgesics. However, an ideal liposome that can evade the host’s immune system while having circulating capability, stability, and no toxicity remains elusive [16]. On the other hand, polymeric nanoparticles may have better stability than liposomal systems, but their biocompatibility and long-term potential safety are issues of concern [17]. Extracellular vesicles (EVs) are membrane-based structures made up of 40–200 nm exosomes or 300–1000 nm microvesicles [18]. They serve as vehicles to carry different types of cellular cargo, such as lipids, proteins, nucleic acids, receptors, and effector molecules, to recipient cells. Exosomes are nanospheres with a membrane bilayer, containing various types of cargo derived from the membrane of the parent cell [19]. In this regard, EVs have many desirable features of an ideal drug delivery system. They have a long, circulating half-life, the intrinsic ability to target tissues, biocompatibility, and minimal or no inherent toxicity, rendering them a superior choice to the majority of the liposomal and polymeric drug delivery systems [20]. EVs have been used to deliver and to enhance the inflammatory activity of the natural product curcumin [21]. Clinical trials have also shown its efficacy and safety in cancer patients [22].

Milk is an evolutionarily successful bioactive fluid containing a fine balance of macro- and micronutrients along with various bioactive factors that act as the complete nutritional package for infants and form a part of the human diet [23]. Previous studies have shown that microRNAs encapsulated in raw milk-derived exosomes enter human circulation and regulate gene expression associated with immunity in peripheral blood cells [24,25]. Moreover, the intestinal transport of raw milk-derived exosomes, mediated by endocytosis in rat primary small intestinal cells and human vascular endothelial cells, also affect gene expression in peripheral blood mononuclear cells. These studies imply that exosomes and EVs can be captured by cellular uptake and passed through the gastrointestinal tract to reach systemic circulation [26].

ALFs not only have in vitro antimicrobial activity and directly kill pathogens but also appear to function in modulating immunity and protecting the host from infection. However, little is known about the functions other than direct killing as an indirect protective role in innate immunity against invasive pathogens. In this study, PT-peptide is derived from anti-lipopolysaccharide factor (PtALF6) of the swimming crab *P. trituberculatus* and contains a putative lipopolysaccharide binding domain.. We investigated the effects of PT-peptide encapsulated in raw milk-derived EVs on immune regulation.

## 2. Results

### 2.1. Characterization of Raw Milk-Derived EVs Encapsulating PT-Peptide 

**Figure 1:**
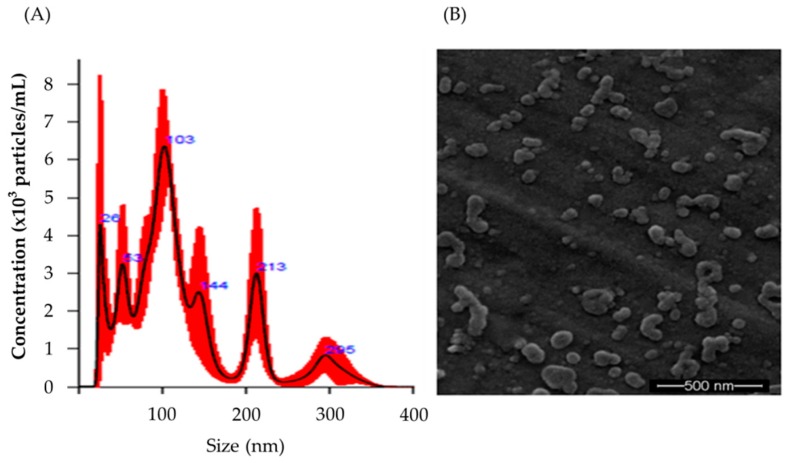
Characterization of raw milk-derived extracellular vesicles encapsulating PT-peptide. The particle size distribution (**A**) and the morphology (**B**) of raw milk-derived EVs encapsulating PT-peptide were determined by NanoSight and scanning electron microscope, respectively.

PT-peptide was encapsulated in raw milk-derived EVs by using freeze-thaw cycles. The particle sizes of the raw milk-derived EVs encapsulating PT-peptide (EVs-PT peptide) ranged from 26 to 295 nm (Figure 1A). The morphology of the EVs-PT peptide observed by scanning electron microscope was similar to exosomes (Figure 1B).

### 2.2. Effect of PT-Peptide and Raw Milk-Derived EVs in THP-1 Cells

**Figure 2:**
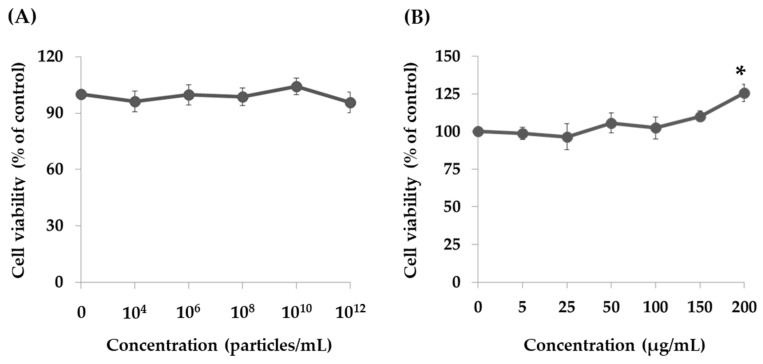
Cell viability of THP-1 cells after treatment with raw milk-derived EVs (**A**) and PT-peptides (**B**) at various concentrations after 24 h treatment. Values were shown as mean ± SD (n = 3). Asterisk (*) shows a significant difference when compared with the control group (*p* < 0.05).

Treating THP-1 cells with 10^4^–10^12^ particles/mL of raw milk-derived EVs for 24 h did not affect cell proliferation, suggesting that raw milk-derived EVs are not cytotoxic to human monocytes (Figure 2A). However, the cell proliferation of THP-1 cells was significantly increased after treatment with 200 μg/mL of PT-peptide for 24 h (Figure 2B). Therefore, PT-peptide could promote the proliferation of THP-1 cells. 

**Figure 3:**
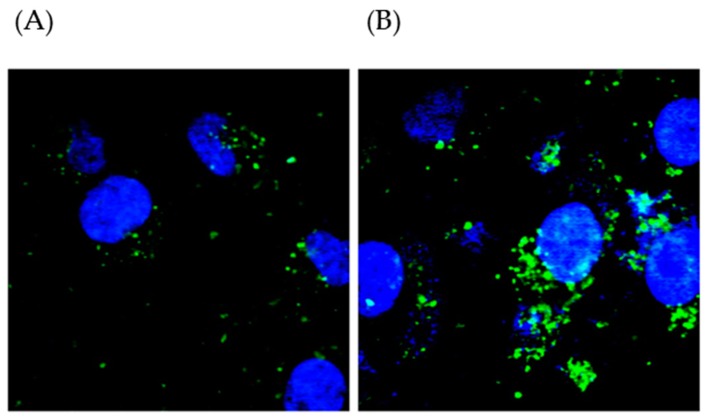
The cellular uptake of PT-peptides in the human THP-1 cells. THP-1 cells were incubated with 200 μg/mL of fluorescein isothiocyanate (FITC)-labeled PT-peptide (**A**) or FITC-labeled PT-peptide encapsulated in raw milk-derived EVs (**B**) for 12 h. After incubation, cells were washed twice and then stained with 4′,6-diamidino-2-phenylindole. The intracellular fluorescence intensity was observed by confocal microscopy. Blue color was DAPI stain and green color was fluorescein isothiocyanate.

Functional peptides are degraded by numerous enzymes within the body that result in dysfunction or loss of bioactivity, and the development of suitable carriers to protect these peptides or proteins is essential. The raw milk-derived EVs are a natural, edible material that could be used to deliver bioactive compounds. We evaluated the efficacy of raw milk-derived EVs in delivering PT-peptides labeled with fluorescein isothiocyanate (FITC) to THP-1 cells. The results showed that fluorescence accumulation in THP-1 cells treated with FITC-labeled PT-peptide encapsulated in raw milk-derived EVs was stronger than those treated with FITC-labeled PT-peptides after 12h incubation (Figure 3). The results suggested that raw milk-derived EVs effectively carried PT-peptides and delivered them to human THP-1 monocytes. Hence, raw milk-derived EVs have the potential to be developed as a delivery system.

### 2.3. Immune Stimulation by PT-Peptide and EVs-PT Peptide in THP-1 Cells

**Figure 4:**
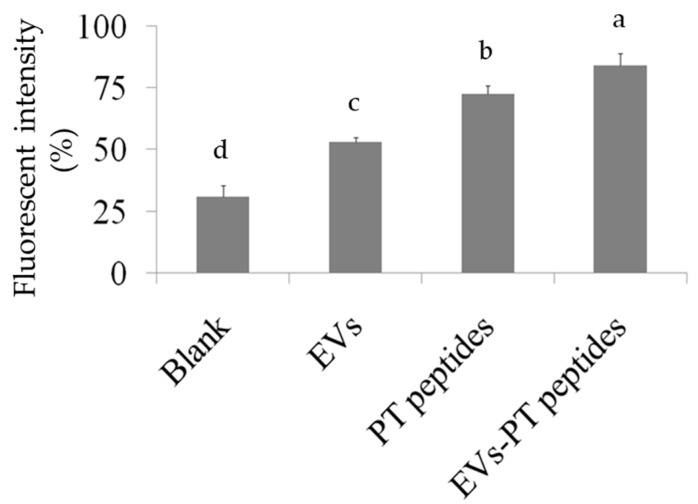
Levels of cellular superoxide production in THP-1 cells. THP-1 cells were treated with 200 μg/mL of raw milk-derived EVs, PT-peptide, or EVs-PT peptide for 24 h. After dichloro-dihydro-fluorescein diacetate staining, the levels of cellular superoxide were assayed by flow cytometry. Different letters represent significant difference (*p* < 0.05).

**Figure 5:**
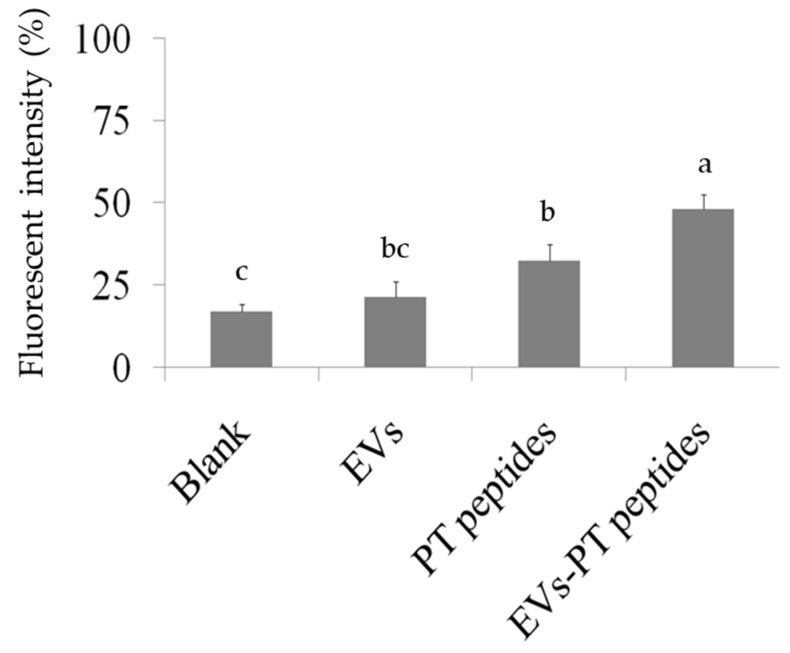
Levels of mitochondrial superoxide production in THP-1 cells. THP-1 cells were treated with 200 μg/mL of raw milk-derived EVs, PT-peptide, or EVs-PT peptide for 24 h. After MitoSox staining, the levels of mitochondrial superoxide were assayed by flow cytometry. Different letters represent significant difference (*p* < 0.05).

Activation and maturation of monocytes by stimulators leads to superoxide anion generation [27]. The levels of cellular and mitochondrial oxidative stress were measured by different staining agents to evaluate the effect of PT-peptide and EVs-PT peptide on oxidative stress in THP-1 cells. For cellular oxidative stress, the dichloro-dihydro-fluorescein diacetate (DCFH-DA) probe was used as an indicator. THP-1 cells generating cellular superoxide were markedly increased after treatment with EVs-PT peptide (84.47%) for 24 h, compared to EVs (53.37%) or PT-peptide (73.27%) treatment only (Figure 4). THP-1 cells after treatment with EVs-PT peptide (48.2%) for 24 h had greater potential for producing mitochondrial superoxide than EVs (21.57%) or PT-peptide (32.17%) alone (Figure 5).

Phagocytosis is a kind of functional index when monocytes are matured by stimulatory trigger [27]. The phagocytic ability of THP-1 cells was 17.35% and 19.7% after treatment with PT-peptide or EVs-PT peptide for 72 h, respectively, whereas phagocytosis was 4.38% upon EVs treatment. The results suggested that PT-peptide activated THP-1 cells and their phagocytic effect were enhanced by encapsulation in EVs.

**Figure 6:**
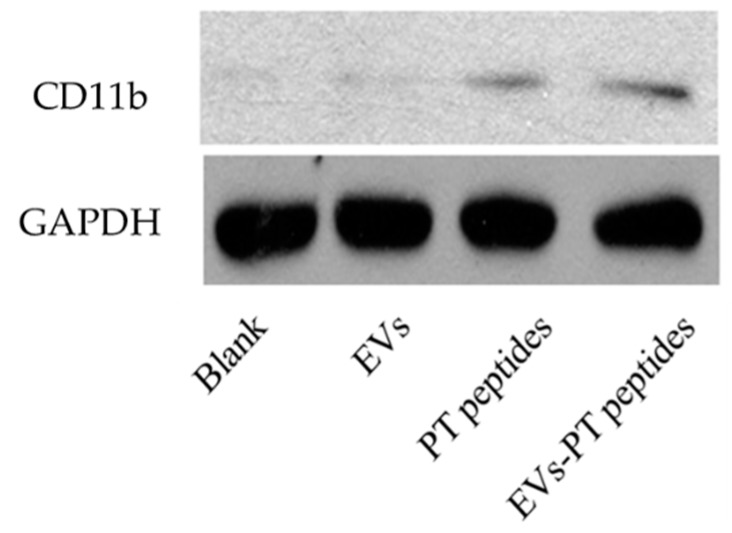
Expression of CD11b in THP-1 cells treated with 200 μg/mL of raw milk-derived EVs, PT-peptide, or EVs-PT peptide for 72 h. Glyceraldehyde 3-phosphate dehydrogenase expression was assessed as an internal reference for normalization.

However, the EVs treatment only promoted cellular oxidative stress, but not mitochondrial oxidative stress and phagocytic ability, suggesting that the function and activity of mitochondria is possibly associated with monocyte maturation. To investigate the maturation of THP-1 cells, their CD marker was determined. Expression of the differentiated marker CD11b was elevated in THP-1 cells after treatment with raw milk-derived EVs, PT-peptide, or EVs-PT peptide for 72 h. However, EVs did not promote the maturation of monocytes, but PT-peptides and EVs-PT peptides could markedly increase the CD11b level in the human THP-1 monocytes (Figure 6). 

**Figure 7:**
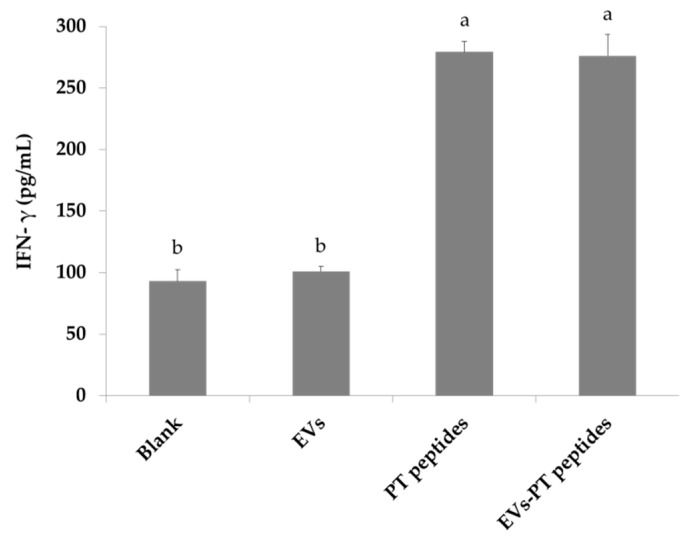
The IFN-γ levels produced from THP-1 cells. THP-1 cells were treated with 200 μg/mL of raw milk-derived EVs, PT-peptide, or EVs-PT peptide for 72 h. The IFN-γ level in culturing medium was determined with an ELISA kit. Values are shown as mean ± SD (n = 3). Different letters represent significant difference (*p* < 0.05).

**Figure 8:**
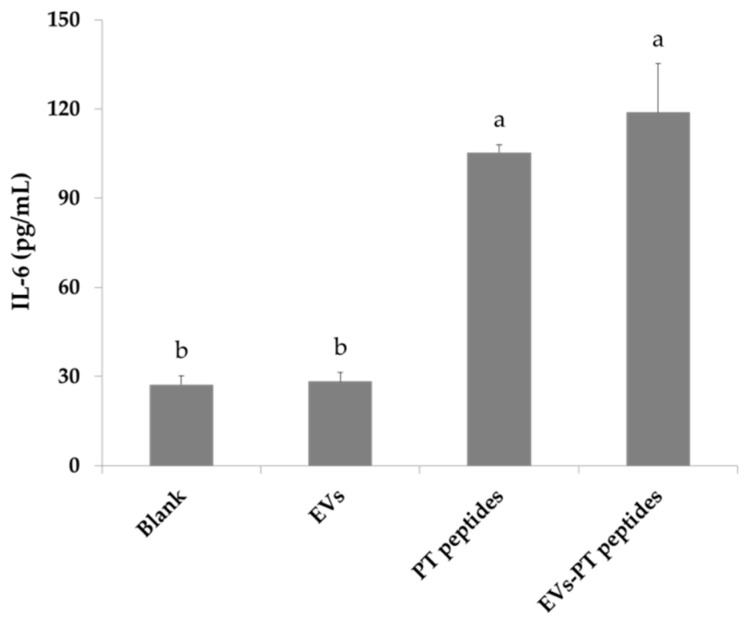
The IL-6 levels produced from THP-1 cells. THP-1 cells were treated with 200 μg/mL of raw milk-derived EVs, PT-peptide, or EVs-PT peptide for 72 h. The IL-6 level in culturing medium was determined with an ELISA kit. Values are shown as mean ± SD (n = 3). Different letters represent significant difference (*p* < 0.05).

**Figure 9:**
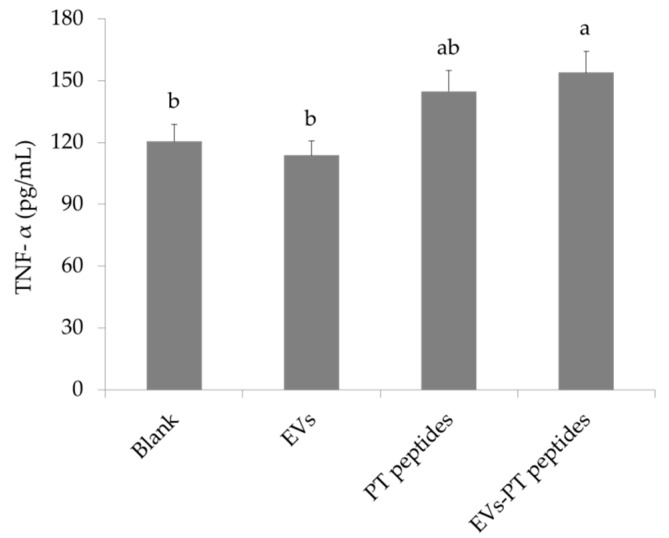
The TNF-α levels produced from THP-1 cells. THP-1 cells (2 × 10^5^ cells/mL) were treated with 200 μg/mL of raw milk-derived EVs, PT-peptide, or EVs-PT peptide for 72 h. The TNF-α level in culturing medium was determined with an ELISA kit. Values are shown as mean ± SD (n = 3). Different letters represent significant difference (*p* < 0.05).

The production of a number of cytokines by matured monocytes, such as tumor necrosis factor-alpha (TNF-alpha) and IL-6, plays an important role in the initiation of the acquired immune response, creating an inflammatory environment favorable for fighting a bacterial infection [28]. The levels of IFN-γ, IL-6, and TNF-α were significantly elevated in THP-1 culture medium after treatment with PT-peptide or EVs-PT peptide for 72 h (Figure 7, Figure 8 and Figure 9). Therefore, PT-peptide could induce the maturation of THP-1 cells.

## 3. Discussion

Depending on the antibacterial mechanism, antimicrobial peptides can be used as substitutes for antibiotics to treat pathogenic bacteria. Cationic AMPs serve as the first chemical barrier between the host organism and the microbe. Their main targets include the cytoplasmic membrane or lipopolysaccharides (LPS) of microorganisms. This response is quick and important, because the activation of pathogen-specific immune responses is slower than the kinetics of microbial proliferation [29]. In this study, PT-peptide is derived from anti-lipopolysaccharide factor (PtALF6) of the swimming crab *P. trituberculatus* and contains lipopolysaccharide binding domain. The results showed that the particle size of EVs ranged from 26 to 295 nm after encapsulating PT-peptide (Figure 1). No cytotoxicity was observed in THP-1 cells treated with EVs for 24 h, whereas PT-peptide significantly promoted cell proliferation of THP-1 cells after 24h treatment (Figure 2), revealing that PT-peptide has potential to regulate immune activity. Furthermore, the increasing immune activity of PT-peptide was enhanced by encapsulation in raw milk-derived EVs (Figure 4 and Figure 5).

The ability of EVs-PT peptide to promote immune activity in THP-1 cells was evaluated. THP-1 cells produce superoxide anion upon activation and maturation by stimulators. Therefore, the intracellular oxidative stress (Figure 4) and mitochondrial oxidative stress (Figure 5) were determined by DCFH-DA and MitoSox staining, respectively. The EVs-PT peptide elevated intracellular superoxide anion in THP-1 cells more than EVs and PT-peptide treatment alone. However, both EVs-PT peptide and PT-peptide treatments markedly increased mitochondrial superoxide anion compared to EVs treatment alone. Similarly, EVs-PT peptide or PT-peptide treatment resulted in a significant increase in the phagocytic ability of THP-1 cells. These results suggest that the production of the mitochondrial superoxide anion plays an important role in monocyte maturation, but not the intracellular superoxide anion.

The functionality of EVs under physiological and pathological conditions relies mostly on what they carry. The application of bovine milk-derived exosomes or EVs as delivery system vehicles for loading chemotherapeutic drugs, functional RNAs, and proteins or peptides has been demonstrated [30]. The cow milk exosomes have been demonstrated to protect microRNAs against harsh digestive processes and to cross the intestinal barrier to reach blood circulation for cellular function [23]. Previous studies have also showed that human milk exosomes were taken up by human intestinal cells [31,32]. In this study, the function of PT-peptide on immune modulation in human THP-1 cells was investigated, and we found that the bovine raw milk-derived EVs elevated the immune activity of the PT-peptide.

Specific expression of CD11b has been found in the initiation of monocytes differentiating into macrophages [27]. In this study, the expression of CD11b was increased in THP-1 cells treated with PT-peptide, suggesting that PT-peptide could induce THP-1 cell differentiation in a specific linkage. Moreover, mature THP-1 cells exerted macrophage-likeability upon PT-peptide or EVs-PT peptide treatment, including superoxide production (Figure 4 and Figure 5), phagocytosis, and secretion of cytokines, including IFN-γ, IL-6, and TNF-α (Figure 7, Figure 8 and Figure 9). The previous study showed that synthetic M-ALF had no direct antimicrobial activity against *Vibrio penaeicida*, whereas ALF-silenced kuruma prawns had significantly higher mortality than untreated prawns after *V. penaeicida* infection [33]. The data suggest that M-ALF plays an indirect protective role against *V. penaeicida* infection and ALF acts as a cytokine-like regulatory molecule, as well as an effector molecule. In this study, the results showed that PT-peptide stimulated THP-1 cells increase secretion of cytokines, including IFN-γ, IL-6, and TNF-α (Figure 7, Figure 8 and Figure 9). Therefore, ALFs not only have antimicrobial activity and directly kill pathogens but also can function as a cytokine-like regulatory molecule.

## 4. Materials and Methods

### 4.1. Materials

PT-peptide (LDHVCNFRVMPRLRSWELYFRGDVWCPGWTVIKG) and FITC-labeled PT-peptide (labeled on N-terminal) were synthesized from LTK BioLaboratories Inc. (Tauyuan, Taiwan). Trypan blue, RPMI 1640 medium, L-glutamine, and sodium pyruvate were purchased from GIBCO (Grand Island, NY, USA). Dichloro-dihydro-fluorescein diacetate (DCFH-DA) and RIPA lysis buffer were purchased from Sigma Chemical Co. (St. Louis, MO, USA). Potassium dihydrogen phosphate (KH_2_PO_4_) and dipotassium hydrogen phosphate (K_2_HPO_4_) were purchased from Merck Co. Ltd.(Darmstadt, Germany). Fetal bovine serum (FBS) was purchased from Hyclone (Logan, UT, USA). GPADH antibody [ab8245] and CD11b antibody [ab133357] were purchased from Abcam (Cambridge, MA, USA).

### 4.2. Preparation of Extracellular Vesicles (EVs)

EVs were isolated using a modification of a previous published method [34]. Briefly, the cells from raw milk were removed by centrifugation at 3000× *g* for 15 min. A differential centrifugation protocol was conducted for 60 min each at 12,000× *g*, 35,000× *g*, and 70,000× *g*. The EVs were pelleted by ultracentrifugation at 100,000× *g* for 60 min, dissolved in 1 mL phosphate buffered saline (PBS), and filtered through 0.22 μm filters to obtain the EVs solution.

### 4.3. Encapsulation and Characterization of EVs

PT-peptide was encapsulated in raw milk-derived EVs, as previously described, with a modification for mixing peptides with EVs using freeze-thaw cycles [35]. Briefly, raw milk-derived EVs were diluted in PBS to a concentration of 0.15 mg/mL total protein. PT-peptide in PBS (0.5 mg/mL) was added to 250 µL of raw milk-derived EVs, and incubated at room temperature for 2 h. The mixture was rapidly frozen to −80 °C and thawed at room temperature. The freeze-thaw cycle was repeated three times. After encapsulation, the particle size and morphology of raw milk-derived EV encapsulating PT-peptide (EVs-PT peptide) were determined using NanoSight™NS500 (Malvern Panalytical) and scanning electron microscope S-2400*SEM* (HITACHI, Tokyo, Japan), respectively.

### 4.4. Cell Culture and Cell Viability Assay

A human monocytic cell line, THP-1, was obtained from the Bioresource Collection and Research Center (BCRC) (Hsinchu, Taiwan). THP-1 cells were cultured in RPMI 1640 medium supplemented with 10% FBS, 2 mM L-glutamine, and 1 mM sodium pyruvate. THP-1 cells (2 × 10^5^/mL) were treated with raw milk-derived EVs (10^4^–10^12^ particles/mL) or PT-peptides (5–200 μg/mL) for 24 h. Cell viability was determined by trypan blue stain [36].

### 4.5. Assay for Cellular Uptake

FITC-labeled PT-peptide was encapsulated in raw milk-derived EVs by freeze-thaw cycle method described in Section 4.3. THP-1 cells (2 × 10^5^/mL) were incubated with 200 μg/mL of FITC-labeled PT-peptide or FITC-labeled PT-peptide encapsulated in raw milk-derived EVs for 12 h. After incubation, cells were washed with PBS twice and then stained with 4′,6-diamidino-2-phenylindole (DAPI). The intracellular fluorescence intensity was observed by confocal microscopy (Leica Microsystems, Mannheim, Germany).

### 4.6. Measurement of Cellular and Mitochondrial Superoxide Levels

Dichloro-dihydro-fluorescein diacetate (DCFH-DA) is commonly used for detecting intracellular oxidative stress [34]. THP-1 cells (2 × 10^5^ cells/mL) were treated with 200 μg/mL of raw milk-derived EVs, PT-peptide, or EVs-PT peptide for 24 h. Oxidative stress was monitored by measuring the level of reactive oxygen species (ROS). The cells were collected and suspended in 500 μL of PBS, mixed with DCFH-DA reagent (final concentration 10 μM) and further incubated for 20 min at 37 °C. The cells were washed thrice with PBS to remove redundant DCFH-DA and resuspended in 500 μL PBS. The cellular oxidative stress was assayed by flow cytometry (Becton-Dickinson, San Jose, CA)

Mitochondrial superoxide indicator MitoSOX™ Red is a fluorogenic dye for highly selective detection of superoxide in the mitochondria of live cells [37,38]. THP-1 cells (2 × 10^5^ cells/mL) were treated with 200 μg/mL of raw milk-derived EVs, PT-peptide, or EVs-PT peptide for 24 h. The cells were stained with 5 μM of MitoSOX™ Red reagent at 37 °C for 30 min and then washed gently three times with PBS. The fluorescence intensity was measured by flow cytometry [37].

### 4.7. Assay for Phagocytosis

In brief, 2 × 10^5^ THP-1 cells were washed with PBS and resuspended in medium after treatment with 200 μg protein/mL of raw milk-derived EVs, PT-peptides, or EVs-PT peptides for 72 h. Then, THP-1 cells were collected and co-cultured with beads-rabbit IgG-FITC complex in medium at 1:200 dilution at 37 °C for two hours. The phagocytosis was observed by microscope after washing with PBS (Becton-Dickinson, San Jose, CA). In addition, the phagocytic ability of THP-1 cells was measured by flow cytometry after cultured with beads-rabbit IgG-FITC complex in medium at 1:200 dilution at 37 °C for two hours.

### 4.8. Western Blotting

THP-1 cells (2 × 10^5^ cells/mL) were treated with 200 μg/mL of raw milk-derived EVs, PT-peptide, or EVs-PT peptide for 72 h. After treatment, the THP-1 cells were rinsed with ice-cold PBS and lysed for 20 min on ice using RIPA lysis buffer containing protease and phosphatase inhibitors. The cells were centrifuged at 12,000 ×g for 10 min at 4 °C. Protein extracts (20 μg) were resolved using 10% SDS-polyacrylamide gel electrophoresis and electrotransferred to polyvinyldiene fluoride membranes (80 V, 120 min). The membranes were blocked with a 5% blocking agent (GE Healthcare Bio-Sciences) in Tris buffered saline containing 0.1% Tween-20 (TBST) for 1 h, and incubated with the primary antibody against Glyceraldehyde 3-phosphate dehydrogenase (GAPDH) (ab8245; 1:2000dilution) or CD11b (ab133357; 1:1000 dilution) overnight at 4 °C. Subsequently, the membranes were washed three times with TBST and incubated with appropriate horseradish peroxidase conjugated secondary antibodies, followed by analysis with an enhanced chemiluminescence (ECL) plus Western blotting detection system (GE Healthcare Bio-Science). GAPDH expression was assessed as an internal reference for normalization.

### 4.9. Assay for Cytokines

THP-1 cells (2 × 10^5^/mL) were treated with 200 μg protein/mL of raw milk-derived EVs, PT-peptides, or EVs-PT peptides for 72 h. Tumor necrosis factor-α (TNF-α), interferon-γ (IFN-γ), and interleukin (IL)-6 levels in culturing medium of THP-1 cells were determined with commercial ELISA kits according to the manufacturer’s instructions (R&D Systems, Minneapolis, MN, USA).

### 4.10. Statistical Analysis

All data were analyzed in triplicates and expressed as means ± standard deviation (SD). One-way analysis of variance (ANOVA) and Duncan’s multiple range tests were carried out. Differences were considered significant when *p* ≤ 0.05. All statistical analyses were performed using SPSS version 13.0 (Chicago, IL, USA). 

## 5. Conclusions

In summary, the results showed that the PT-peptide activated THP-1 cells through producing mitochondrial superoxide anion and enhancing phagocytic ability, leading to maturation of monocytes and secretion of cytokines, which play important roles in innate and adaptive immunity. PT-peptide may be a useful therapeutic agent in humans, as it is not toxic to cells and induces increases in immunity. These results suggest that PT-peptide has the potential to be developed as an immune stimulator.
